# 851. Rate of Sexually Transmitted Infections and Engagement in HIV Pre-Exposure Prophylaxis at the Veterans Affair Maryland Health System

**DOI:** 10.1093/ofid/ofab466.1046

**Published:** 2021-12-04

**Authors:** Omar Harfouch, Emily Comstock, Roman Kaplan, Rohit Talwani, Eleanor Wilson

**Affiliations:** 1 University of Maryland Medical Center, Division of Infectious Diseases, Baltimore, Maryland; 2 Veterans Affairs Maryland Health Care System, Baltimore, Maryland; 3 Baltimore VA Medical Healthcare Center, Baltimore, Maryland; 4 University of Maryland School of Medicine, Baltimore VA, Baltimore, Maryland; 5 Institute of Human Virology, University of Maryland School of Medicine, Baltimore, Maryland

## Abstract

**Background:**

Rates of sexually transmitted infections (STIs) and uptake of HIV pre-exposure prophylaxis (PrEP) during the 2020 coronavirus pandemic are unknown. We evaluated data from the Veterans Affair Maryland Health Care System (VAMHCS) data to determine rates of STI and PrEP linkage in our Veterans.

**Methods:**

We extracted patient-level data on demographics, STI testing (chlamydia, gonorrhea, and syphilis), International Classification of Diseases (ICD) diagnosis codes and refills of TDF-FTC and TAF-FTC. We compared the ratio of positive STI tests in 2018, 2019 and 2020 using chi-square tests. Individuals eligible for PrEP were defined as patients with a newly positive STI result or an ICD diagnosis of: high risk sexual behavior; an STI mentioned above; or gender identity disorder. We excluded anyone with a positive HIV test or a creatinine >1.8. We identified patients initiated on PrEP through pharmacy refill data to define initiation of care. Finally, we used chi-square tests to compare differences of initiation of PrEP between years and demographics.

**Results:**

The STI positivity rate significantly increased (p< 0.01) from 44.2% (2018) and 42.9% (2019) to 61.6% (2020) [Table 1]. The median ages of those who had a positive STI test were 50 (2018), 44 (2019) and 44 (2020). In 2020, 17% of patients eligible for PrEP filled PrEP. Engagement was similar (p=0.33) in 2019 and 2018, where 14% and 11.6% of patients eligible for PrEP received a prescription (p-value=0.33) [Figure 1]. The median age of those refilling PrEP were: 44 (2018); 43 (2019) and 41 (2020)). In 2020, we observed a statistically significant difference (p< 0.01) in initiation of PrEP in care among Black patients with 11.7% of eligible patients filling PrEP as compared to white patients (26.2%) and other races (23.3%) [Figure 2].

Table 1. Rate of positive tests at VAMHCS from 2018-2020.

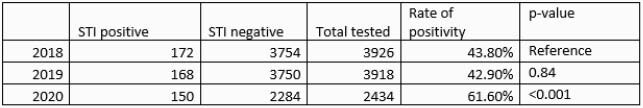

Figure 1. PrEP Cascade at VAMHCS by year. Non-statistically significant (P=0.33) when comparing engagement in care between different years.

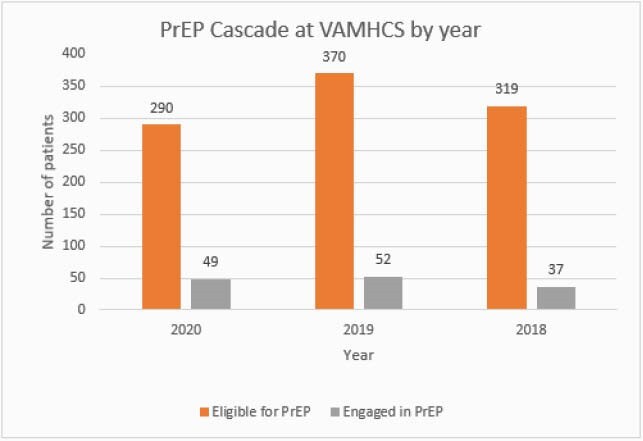

Figure 2. Racial distribution of PrEP eligibility and initiation by year at the VAMHCS.

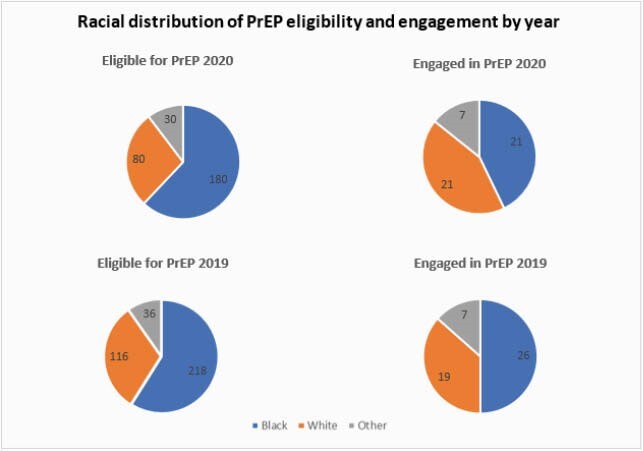

**Conclusion:**

While during the coronavirus pandemic in 2020, fewer Veterans sought STI testing at the VAMHCS, the number of positive STI results remained steady, leading to a higher positivity rate. The rate of initiation of PrEP did not differ between 2020, 2019 and 2018. Racial inequities in initiation of PrEP increased in 2020.

**Disclosures:**

**All Authors**: No reported disclosures

